# First Complete Genome Sequence of *Pseudomonas aeruginosa* (Schroeter 1872) Migula 1900 (DSM 50071^T^), Determined Using PacBio Single-Molecule Real-Time Technology

**DOI:** 10.1128/genomeA.00932-15

**Published:** 2015-08-20

**Authors:** Kazuma Nakano, Yasunobu Terabayashi, Akino Shiroma, Makiko Shimoji, Hinako Tamotsu, Noriko Ashimine, Shun Ohki, Misuzu Shinzato, Kuniko Teruya, Kazuhito Satou, Takashi Hirano

**Affiliations:** Okinawa Institute of Advanced Sciences, Uruma, Okinawa, Japan

## Abstract

The first complete genome sequence of the type strain *Pseudomonas aeruginosa* (Schroeter 1872) Migula 1900 (DSM 50071^T^) was determined in a single contig by PacBio RS II. The genome (6,317,050 bp, G+C content of 66.52%) contained 10 sets of >1,000-bp identical sequence pairs and 183 tandem repeats.

## GENOME ANNOUNCEMENT

*Pseudomonas aeruginosa* is an aerobic, motile, and Gram-negative rod-shaped bacterium that exists in a wide range of ecological niches (1–4). It is a major opportunistic human pathogen and is also an important causative agent of hospital-acquired nosocomial infections, characteristically in immunocompromised individuals (1–4). The emergence of antibiotic-resistant forms of *P. aeruginosa* is a worldwide problem in clinical medicine (5, 6). For instance, a total of 161 clinical isolates of multidrug-resistant *P. aeruginosa* were obtained between July and September 2011 from 161 hospitals in 30 of 47 prefectures in Japan, where two novel IMP-type metallo-β-lactamase variants were identified (7). *P. aeruginosa* can develop resistance to antibiotics through either acquisition of resistance genes on mobile genetic elements or mutational processes that alter the expression and/or function of chromosomally encoded mechanisms (5).

The type strain of *P. aeruginosa* (Schroeter 1872) Migula 1900 (DSM 50071^T^) was first reported in 1872 by Schroeter (8–11). At present, more than 20 complete genome sequences of *P. aeruginosa* are publicly available (http://www.ncbi.nlm.nih.gov/genome/genomes/187). *P. aeruginosa* has a genome size of around 6.3 Mb, with a G+C content of around 66.6% (2). The type strain is usually the first isolated strain of the species and exhibits all of the relevant genotypic and phenotypic properties cited in the species circumscriptions; therefore, the complete genome sequence of the type strain is crucial to analyzing, comparing, and evaluating the characteristics of the species (12). Here, we report the first complete genome sequence of *P. aeruginosa* DSM 50071^T^ determined by single-molecule real-time (SMRT) technology (13).

Genomic DNA obtained from Deutsche Sammlung von Mikroorganismen und Zellkulturen (DSMZ) was purified using the PowerClean DNA cleanup kit (MO BIO Laboratories, Carlsbad, CA), followed by 20-kb library construction for P5-C3 chemistry without shearing. After size selection of the upper 7 kb by BluePippin (Sage Science, Beverly, MA), 16 SMRT cells of the libraries were sequenced on the PacBio RS II platform (Pacific Biosciences, Menlo Park, CA) with 180-min movies. *De novo* assembly was performed using the hierarchical genome assembly process 3 (HGAP3) workflow (14). A single circular contig representing a chromosome was obtained (6,317,050 bp, average G+C content of 66.52%, and 843× coverage). The genome contained 10 sets of >1,000-bp identical sequence pairs (5,288 bp maximum) and 183 tandem repeats (246 bp × 20.7 copies maximum). On DSM 50071^T^ sequencing, the PacBio RS II platform produced extra-long reads with an average of 6,256 bp and a maximum of 28,135 bp. The SMRT technology provides power for genome sequencing with extra-long multikilobase reads and unbiased G+C coverage (15), thereby resolving these hard-to-sequence regions.

The complete genome sequence of the *P. aeruginosa* type strain reported here can be used as the standard reference for the species and will accelerate the understanding of the pathogenomic characteristics of the species, especially in (antibiotic-resistant) *Pseudomonas* infection.

### Nucleotide sequence accession number.

The complete genome sequence of *P. aeruginosa* DSM 50071^T^ has been deposited in DDBJ/ENA/GenBank under the accession number CP012001.
